# Occurrence and potential implications of the hook effect in a *Borrelia burgdorferi* IgG antibody chemiluminescence immunoassay

**DOI:** 10.1007/s10096-025-05172-y

**Published:** 2025-05-21

**Authors:** Marc Westerholt, Torbjörn Kjerstadius, Lukas Frans Ocias

**Affiliations:** 1 Department of Clinical Microbiology, Karlstad Hospital, Karlstad, Sweden; 2 Centre for Clinical Research and Education, Region Värmland, Karlstad, Sweden; 3 Department of Biomedical and Clinical Sciences, Linköping University, Linköping, Sweden; 4 School of Medical Science, Faculty of Medicine and Health, Örebro University, Örebro, Sweden

**Keywords:** Prozone phenomenon, *Borrelia* serology, IgG antibodies, Lyme disease diagnostics, Immunoassay optimization, Dilution recommendation, Diasorin, Liaison *Borrelia* IgG immunoassay, Overdilution, Dilution threshold

## Abstract

**Purpose:**

Lyme borreliosis is diagnosed based on clinical symptoms and serological analysis, with high IgG antibody levels associated with late manifestations. Immunoassays with a broad detection range can exhibit a hook effect, leading to false-low results and potential misdiagnosis. This study aimed to investigate the occurrence of the high-dose hook effect and determine a dilution threshold to detect the hook effect and avoid overdilutions for the Liaison *Borrelia* IgG immunoassay.

**Methods:**

In a two-year period, 5639 patient samples analysed for *Borrelia* antibodies were screened at Karlstad Hospital, Sweden. Samples with IgG ≥ 75 AU/mL and < 75 AU/mL with detectable IgM underwent 1:10 dilution, with a further 1:50 dilution for samples with IgG > 240 AU/mL. A Gaussian Mixture Model was used to group samples with and without hook effect.

**Results:**

Of 389 samples eligible for dilution, 262 with IgG < 240 AU/mL were analysed. Of these, 70 (26.7%) showed hook effect, corresponding to 18% of included and 1.2% of all screened samples. Overdilution occurred in 58 (22.1%) diluted samples. Dilution thresholds of 98.7 and 96 AU/mL detected 95% of hook effect and overdilution samples, respectively.

**Conclusion:**

A substantial number of samples showed hook effect, which could lead to missed late Lyme borreliosis manifestations and inaccurate intrathecal index calculations. A dilution threshold of 98.7 AU/mL in the Liaison *Borrelia* IgG immunoassay effectively identified 95% of hook effect and avoided more than 95% of overdilutions.

## Introduction

The diagnosis of Lyme borreliosis (LB) rests on a combination of clinical manifestations and the detection of *Borrelia burgdorferi* sensu lato (*Bbsl*)-specific antibodies [1]. *Bbsl*-specific IgG antibody levels are often interpreted semiquantitatively as being reflective of disease progression but should always be related to the clinical manifestations. High IgG antibody levels are associated with late manifestations such as arthritis, acrodermatitis chronica atrophicans and some cases of late neuroborreliosis [2]. Immunoassays used for the diagnosis of LB should therefore ideally cover a wide detection range to identify patients with late-stage infection. Prozone or hook effect, named after the hook-shaped dose-response like curve in immunoassays, occurs at high analyte concentrations. High analyte levels, of either antibodies or antigens, bind to conjugate and detector antibodies, thus inhibiting the formation of immunocomplexes and leading to false-low results and potential misdiagnosis. This phenomenon has been a recurrent pitfall in a variety of immunoassays with a wide detection range e.g. rapid plasma reagin (RPR)/Veneral Disease Research Laboratory test (VDRL), cryptococcal antigen and malaria antigen and is usually handled by diluting the sample or increasing the quantity of antigen or antibodies in the test depending on the analyte [3, 4, 5, 6]. In *Bbsl* assays, the hook effect can result in both false-positive and false-negative intrathecal antibody index calculations and high antibody levels may be missed in patients with late manifestations.

The Liaison *Borrelia* IgG chemiluminescence immunoassay (CLIA) (Diasorin, Saluggia, Italy) is among the widest adopted *Bbsl* screening assays in Sweden and the Nordic countries. In November 2021, the Swedish national reference laboratory for *Borrelia* diagnostics issued a warning regarding false-low results in this *Bbsl* immunoassay, which could be resolved through dilution. Thus far, the manufacturer recommends dilution at IgG levels above 240 AU/mL.

The extent of the hook effect is currently unclear, and a reasonable dilution threshold that identifies the hook effect and avoids dilution to the point of undetectability (overdilution) remains to be determined. The aim of this study was to investigate the occurrence and affected range of high-dose hook effect and to determine a dilution threshold to detect hook effect and avoid overdilutions in the Liaison *Borrelia* IgG immunoassay.

## Materials and methods

**Fig. 1:**
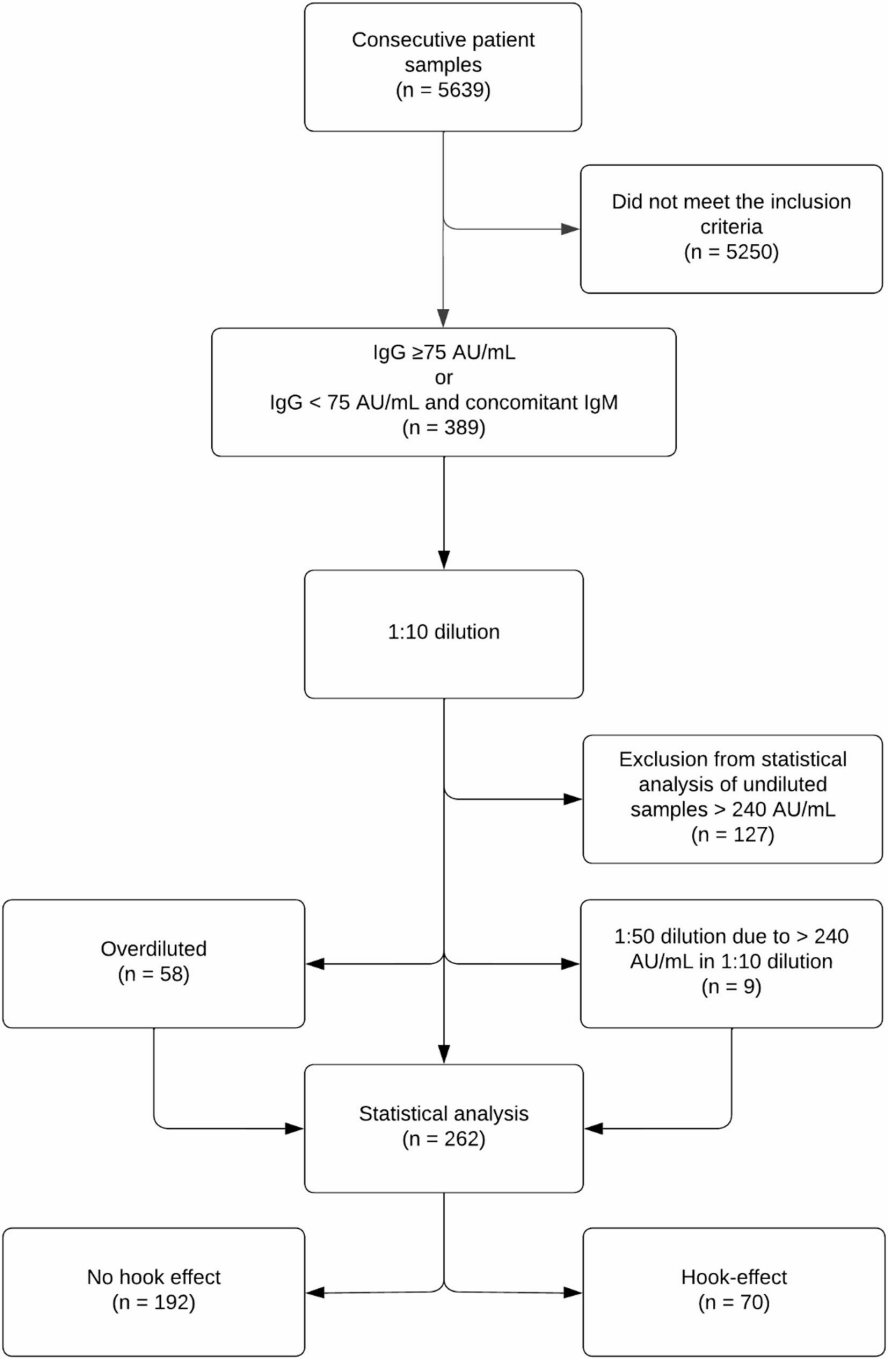
Flowchart of the inclusion, dilution and exclusion of samples

The study population of 5639 consecutive patient samples analysed for *Bbsl* antibodies at the department of Clinical Microbiology at Karlstad Hospital, Sweden, was screened for eligibility in the period 14-04-2021 to 28-02-2023. Samples with IgG levels ≥ 75 AU/mL or < 75 AU/mL with concomitantly detectable IgM were included and underwent dilution. Samples underwent 1:10 dilution and further 1:50 dilution if IgG levels were > 240 AU/mL in the 1:10 dilution. Results before dilution and the adjusted results after dilution were manually annotated by the laboratory technician and later used in the calculations. A total of 389 samples (6.8%) were included and underwent dilution (Fig. 1). Of these, 127 (32.6%) had an IgG reactivity > 240 AU/mL in the undiluted sample and were excluded as the manufacturer already recommends dilution above this level, leaving 262 samples (222 samples with ≥ 75 AU/mL and 40 samples with < 75 AU/mL and concomitant IgM) for further statistical analysis.

### Laboratory analysis

The Liaison *Borrelia* IgG and IgM immunoassays were used (assay number: 310880 and 310020) on the Liaison XL platform. The manufacturer recommends dilution at IgG levels > 240 AU/mL. For this study, samples meeting the inclusion criteria were diluted.

### Data analysis

Serum samples were divided into two clusters (with and without hook effect) using a Gaussian Mixture Model (GMM). The model was applied to the percentage change in reactivity between diluted and undiluted sample:

$$\displaylines{\:Change = \: \cr \frac{{\displaylines{Reactivity}\:in\:diluted\:sample - \displaylines{Reactivity}\:in\:undiluted\:sample}}{{\displaylines{Reactivity}\:in\:undiluted\:sample}}\: \cr \times \:100 \cr} $$

The data were modelled as a mixture of two Gaussian components corresponding to samples with and without hook effect. Model parameters (including means, variances and mixture weights) were estimated with the Expectation-Maximization algorithm. Samples were clustered based on the posterior probability of belonging to each Gaussian component. The analysis was performed using the “mclust” R package [7].

Overdilutions were included to evaluate the cost-effectiveness of diluting the samples and were defined as diluted samples (1:10 and 1:50), which received an overdilution warning on the Liaison XL instrument. Overdiluted samples inherited the undiluted result in the statistical analysis. Samples that underwent 1:10 and 1:50 dilutions were grouped together as not all samples underwent both dilutions.

The 5th percentile was calculated for included samples in the hook effect cluster to determine a level which would identify 95% of samples with a hook effect.

The 95th percentile was calculated for included samples with an overdilution to determine a level which would prevent 95% of overdilutions.

Segmented linear regression was used to detect changes in the relationship between reactivity in the diluted and undiluted sample. The regression was conducted on the entire dataset including both groups. A preliminary linear regression for undiluted reactivity was performed and then segmented to identify a breakpoint for the change in relationship between diluted and undiluted reactivity. The breakpoint was estimated using an iterative least squares approach. Since not all samples exhibited a hook effect, only the presence or absence of a breakpoint was considered, rather than specific model parameters. The analysis was performed using the “segmented” R package [8].

A modified Bland-Altman plot was created to visualise differences in reactivity and affected range between diluted and undiluted samples as a function of the reactivity in the undiluted samples.

All statistical analyses and the Bland-Altman plot were performed using R version 4.2.1 [9].

### Ethical considerations

The study was approved by the Swedish Ethical Review Authority (reference: 2023-03116-02).

## Results

**Fig. 2:**
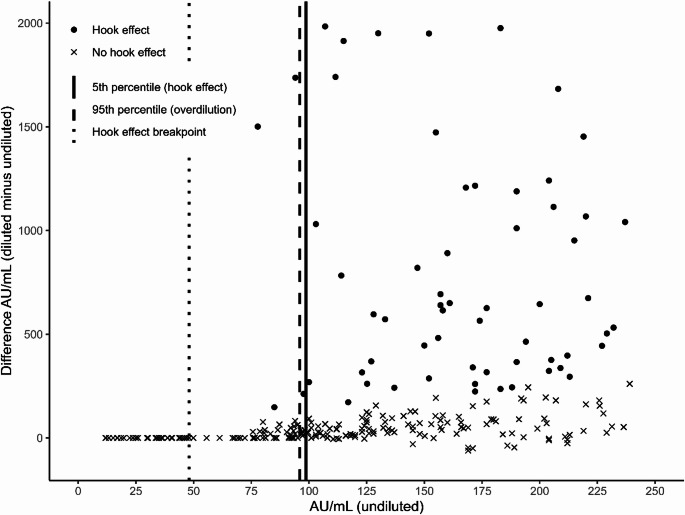
A modified Bland-Altman plot, showing increasing variability in the difference between diluted and undiluted samples with increasing IgG antibody level in the undiluted samples. Samples belonging to the hook effect cluster are marked with dots. Samples in the no hook effect cluster are marked with crosses. The 5th percentile for samples with hook effect, 95th percentile for overdiluted samples and the breakpoint from linear segmented regression are marked with full, dashed and dotted line, respectively

The undiluted samples displayed a range of 11.82–239 AU/mL and had a median reactivity (interquartile range) of 126 AU/mL (79.15 AU/mL). For the diluted samples the range and median reactivity (interquartile range) was 11.82–7434 AU/mL and 189.6 AU/mL (296.2 AU/mL), respectively. Of the 262 samples, which underwent dilution, 70 (26.7%) were assigned to the hook effect cluster corresponding to 18% of included samples (*n* = 389) and 1.2% of all screened samples (*n* = 5639) (Fig. 2). No hook effect was observed in 192 samples, of which 58 (22.1%) were overdiluted (Fig. 1). A 5th percentile of 98.7 AU/mL of undiluted sample was calculated for samples with hook effect and a 95th percentile of 96.0 AU/mL of undiluted sample was calculated for the overdiluted samples (Fig. 2).

The lowest change in reactivity categorised as exhibiting the hook effect by the GMM was an increase of 129%. Although the hook effect cluster was right skewed, the model had a clear classification with negligible overlap between the groups, and the assumption of two gaussian components was supported by the Bayesian Information Criterion. A breakpoint of 48 AU/mL with a confidence interval of 48–108 AU/mL was estimated with segmented linear regression (Fig. 2). Only overdilutions occurred below the breakpoint, whereas 28 of the 58 overdilutions (48.3%) were observed above the breakpoint.

The modified Bland-Altman plot showed an increasing variability in the difference between diluted and undiluted samples with increasing IgG antibody level in the undiluted sample. This increasing variability was observed at levels 77.8–239 AU/mL in the undiluted samples (Fig. 2).

A double hook effect, i.e. two consecutive hook effects in the undiluted and first diluted sample, was observed in one sample. This sample displayed an initial IgG level of 119.5 AU/mL, which increased to 1979 AU/mL and 2710 AU/mL, respectively following 1:10 and 1:50 dilution.

## Discussion

This is the first study describing a high-dose hook effect in the Liaison *Borrelia* IgG immunoassay. The hook effect was primarily observed in undiluted samples with a reactivity of > 98.7 AU/mL. Despite this, a hook effect could still be observed in a few samples with a reactivity of < 98.7 AU/mL. The lowest level at which a hook effect was observed was 77.8 AU/mL, which, following dilution, increased to 1579 AU/mL. The breakpoint of 48 AU/mL, as estimated by the segmented linear regression, represents the lowest theoretical value at which the hook effect is predicted to occur. However, many of the observed overdilutions (48.3%) occurred above the breakpoint making it an unfeasible dilution threshold. The lowest observed hook effect was within the confidence interval of the linear segmented regression (48–108 AU/mL), corroborating that the hook effect can occur within this interval. Thus, caution must be exercised when interpreting IgG antibody results in patients strongly suspected of late manifestations of LB even at these lower levels. Of note, a double hook effect was observed when a sample was reanalysed. This is not surprising since every diluted sample result is multiplied by the dilution factor and is therefore just as vulnerable to the hook effect as undiluted samples. Diluted samples with results in the span between 98.7 AU/mL and 240 AU/mL (values before multiplying with the dilution factor) should therefore be considered for further dilution since the IgG reactivity of the diluted sample can still be high enough to cause a hook effect.

Prior studies on the hook effect within clinical microbiology have focused on flocculation tests or lateral flow assays, which typically lead to false negative results [3, 4, 5, 6]. The hook effect has also been described in assays detecting albumin and a wide range of tumor markers [10]. In general, the vast majority of assays with hook effect interferences are direct immunoassays and can lead to both false negative and false low results. Indirect immunoassays, however, are in general less sensitive to the hook effect but can still lead to false low results [10]. The Liaison *Borrelia* IgG assay is an indirect immunoassay displaying hook effect, which is uncommon.

The hook effect entails several clinical implications for patients under investigation for LB. When present in serum, it can lead to late manifestations of LB, characterised by high IgG levels, being missed [1]. However, even though this study investigated serum samples, it is likely that the hook effect also applies to cerebrospinal fluid (CSF), as the hook effect is a limitation of the immunoassay, not the type of sample. A similar dilution threshold of 98.7 AU/mL is therefore warranted for CSF until the hook effect has been further investigated. In this context, a hook effect could impact the calculation of the intrathecal antibody index, as a false-low result in CSF can lead to a false-negative index, which may result in a missed diagnosis of neuroborreliosis and potential undertreatment [1, 11]. Conversely, a false-positive index can also occur if the serum sample alone displays a hook effect, resulting in possible misdiagnosis and unnecessary antibiotic treatment. No studies have reported a hook effect in *Bbsl* IgM assays, but such a finding would warrant similar precautions when calculating the intrathecal antibody index.

The inclusion criteria (≥ 75 AU/mL and < 75 AU/mL with concomitant IgM) were based on our experience that the hook effect is mainly observed in the upper range. As overdilutions are overrepresented in the lower range of the immunoassay, we used the presence of IgM reactivity ≥ 75 AU/mL to select samples for dilution, thereby limiting unnecessary overdilutions. These criteria were a pragmatic approach to reduce the time and costs of the study but may have excluded some patients with late manifestations in the lower range without detectable IgM reactivity. No hook effect was observed in the 45 samples displaying a reactivity below 77.8 AU/mL in the undiluted samples and it is therefore unlikely that the inclusion criteria have introduced a substantial selection bias. A single dilution group, which included both 1:10 and 1:50 dilutions, was used because the 1:50 dilution group was too small for any subgroup analysis but was necessary for the descriptive statistics due to truncated values (> 240 AU/mL) in the 1:10 dilution group.

The lowest change in reactivity categorised as the hook effect cluster was an 129% increase and the GMM can therefore be considered relatively conservative since the methodological variation is significantly lower [12]. The segmented linear regression may underestimate the real breakpoint since not all samples display a hook effect.

Whether a hook effect also affects IgM antibodies or is also present in other immunoassays detecting *Bbsl* IgG antibodies is currently unknown and should be subject to further investigation. In addition, studies investigating the hook effect in CSF are warranted.

In conclusion we detected a hook effect in a substantial number of samples, which could lead to late manifestations of LB, often presenting with high IgG levels, being missed. Furthermore, the hook effect can lead to both false-positive and false-negative intrathecal index calculations in patients suspected of neuroborreliosis. Thus, a dilution threshold of > 240 AU/mL, as recommended by the manufacturer, is inadequate. Instead, a dilution threshold of 98.7 AU/mL for the analysis of *Bbsl* IgG antibodies using the Liaison *Borrelia* IgG immunoassay is reasonable. Despite this, caution is warranted, even at IgG values below this threshold, when testing patients suspected of late manifestations of LB.

## Data Availability

The datasets used and/or analysed during the current study are available from the corresponding author on reasonable request.
